# Magnetic Nickel‐Containing Heterogeneous Catalysts for the Heck Reaction: Catalyst Design, Performance, and Sustainability

**DOI:** 10.1002/open.70244

**Published:** 2026-06-17

**Authors:** Forouzan Karimi, Hamideh Sarreshtehdar Aslaheh, Ahmad Poursattar Marjani, Kurosh Rad‐Moghadam

**Affiliations:** ^1^ Department of Organic Chemistry Faculty of Chemistry University of Guilan Rasht Iran; ^2^ Department of Organic Chemistry Faculty of Chemistry Urmia University Urmia Iran

**Keywords:** Heck reaction, Ni‐based catalysts, transition metal catalysis

## Abstract

Heck reaction is one of the key processes for forming carbon–carbon bonds in modern organic synthesis and is typically catalyzed by palladium complexes. In recent years, considerable attention has been devoted to the use of nickel as a cocatalyst/modifier alongside palladium as well as the active component in hybrid systems; in particular, magnetic nickel‐based nanocatalysts, owing to their high specific surface area, good tolerance toward various functional groups, and easy recovery using a magnetic field, are considered efficient and recyclable options for palladium‐catalyzed Heck reactions. This review summarizes recent advances in the design, synthesis, and application of nickel‐based magnetic nanocatalysts, with a focus on systems combined with palladium (including nanocomposites, Ni–Pd bimetallic structures, and supported hybrid nanostructures) in the Heck reaction. In many cases, the highlighted catalysts exhibit high yields and desirable recyclability under mild conditions (including aqueous or solvent‐free media). Strategies such as surface functionalization, the use of synergistic supports like graphene oxide, silica, and carbon nanotubes, and the engineering of bimetallic/multimetallic systems (to enhance performance alongside palladium) are also discussed. This review covers studies from 2013 to 2025.

## Graphical abstract

This schematic outlines a general Heck coupling using magnetic nickel‐based catalysts. The reactants are mixed and heated to promote carbon–carbon bond formation and produce a substituted alkene product. After completion, an external magnet enables rapid separation of the nickel catalyst from the reaction mixture. This approach simplifies workup, improves catalyst recovery, and supports potential reuse in subsequent reaction cycles.

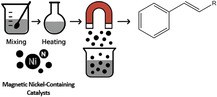

**Abbreviations**
β‐CD: β‐cyclodextrin
CNTs: carbon nanotubes
DMF: *N*,*N*‐dimethylformamide
EDTA: ethylenediaminetetraacetic acid
EG: ethylene glycol
Et3N: triethylamine
FESEM: scanning electron microscopy
FTIR: fourier transform infrared
GC: gas chromatography
GO: graphene oxide
ICP‐OES: inductively coupled plasma optical emission spectrometry
Ni‐MNPCD: nickel supported on β‐cyclodextrin‐functionalized magnetic nanoparticles
NSAID: nonsteroidal anti‐inflammatory drugs
OLED: organic light‐emitting diodes
rGO: reduced graphene oxide
TCT: 2,4,6‐trichloro‐1,3,5‐triazine
TEM: transmission electron microscopy
TGA: thermogravimetric analysis
UV–Vis: ultraviolet/visible spectroscopy
VSM: vibrating‐sample magnetometer
WO3: tungsten trioxide
XRD: X‐ray diffraction
XPS: X‐ray photoelectron spectroscopy.

## 1 Introduction

**FIGURE 1:**
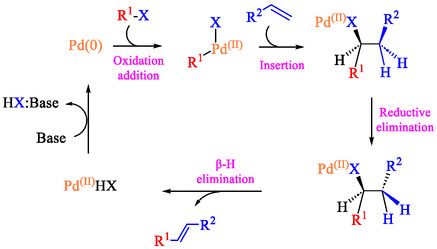
The mechanism of the Heck reaction.

The Heck reaction, so‐called because of the work of chemist Richard F. Heck, is a crucial carbon–carbon coupling reaction in organic chemistry, recognized for its precision and efficiency in forming complex molecules [1]. It is a class of reaction where aryl halides react with alkenes under palladium catalysts to form substituted alkenes. This reaction is also renowned for its capacity to form carbon–carbon bonds, an essential process in organic compound synthesis [2]. Heck reaction is not just about synthesizing low‐molecular‐weight molecules; it is necessary in the production of drugs, agrochemicals, and organic materials, hence equally vital in industry and research. A clear understanding of the mechanism of the Heck reaction shows a stepwise process (Figure 1). These steps are facilitated by transition metal catalysts, mainly palladium, to couple the aryl halides and alkenes selectively and efficiently. The reaction may be carried out under mild conditions and is efficient on all classes of functional groups; thus, it is an all‐purpose tool for chemists to facilitate the compact synthesis of intricate molecular structures [3]. At the core of the Heck reaction is a series of steps wherein an aryl halide adds to a palladium catalyst, followed by the insertion of an alkene into the palladium–aryl bond, and culminating in a reductive elimination that yields the desired product and regenerates the catalyst [4]. The significance of the Heck reaction lies in its ability to preserve stereochemical configuration and achieve high yields, making it a reaction of choice for chemists seeking to synthesize intricate organic frameworks efficiently [5, 6, 7]. In Figure 1, the standard mechanism of this reaction using a palladium catalyst is presented.

**FIGURE 2:**
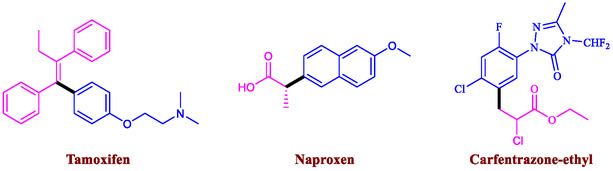
Chemical structures of key compounds: tamoxifen, naproxen, and carfentrazone‐ethyl were synthesized using the Heck reaction for applications in pharmaceuticals and agrochemicals.

Figure 2 presents the chemical structures of the major pharmaceutical and agrochemical compounds, tamoxifen [5], naproxen [8], and carfentrazone‐ethyl [6], prepared through the Heck reaction. Tamoxifen, as an agonist of estrogen receptors used to treat breast cancer, is prepared through the Heck reaction by coupling 4‐bromo‐*N*,*N*‐dimethylaniline and styrene. Naproxen, an over‐the‐counter medication that is an NSAID employed to treat pain as well as inflammation, is prepared via Heck coupling reactions between acrylic acid derivatives and aryl halides. Carfentrazone‐ethyl, a herbicide, is prepared via aryl halide and acrylate ester coupling. Lastly, all this indicates the merit of Heck reactions as a route to fairly complex molecules of chemical and biological importance and the potential applications of surface‐functionalized magnetic nickel‐based nanocatalysts to the sustainability and cost‐effectiveness of traditional palladium catalysts in organic synthesis.

Catalysis, or the acceleration of a chemical reaction through the addition of a catalyst, is a key concept behind many chemical processes. Catalysts present an alternate reaction pathway with lower activation energy, leading to faster reactions without being consumed in the reaction. For the Heck reaction, the catalyst enables the breaking and forming of bonds between aryl halides and alkenes, which are necessary for synthesizing target molecules [7]. Although the Heck reaction has long been using palladium, recent research has started examining the possibility of using other transition metals like nickel as cocatalysts [8, 9].

### 1.1 Features of Magnetic Ni Nanoparticles

Magnetic nickel (Ni) nanocatalysts have garnered tremendous attention in recent years as superb and eco‐friendly catalysts in various organic transformations. The importance of magnetic Ni nanocatalysts is due to several reasons:

#### 1.1.1 Abundance and Cost‐Effectiveness

Nickel is an earth‐abundant, low‐cost transition metal compared to precious metals like palladium, platinum, or rhodium. This makes Ni‐based catalysts cost‐effective for large‐scale industrial applications [10].

#### 1.1.2 Magnetic Properties

The ferromagnetic nature of Ni nanoparticles in itself facilitates easy separation of the catalyst from reaction mixtures by a mere external magnetic field. Such a feature greatly eases catalyst recovery and reuse, reducing waste and process expense [11, 12].

#### 1.1.3 High Catalytic Activity

Ni nanoparticles exhibit high catalytic activity and selectivity in a broad spectrum of critical organic reactions due to their very high surface area, tunable electronic states, and ability to activate a wide range of chemical bonds.

#### 1.1.4 Eco‐Friendly and Mild Reaction Conditions

The majority of Ni nanocatalysts work well at mild, often aqueous or solvent‐free conditions, consistent with green chemistry principles [13].

#### 1.1.5 Cross‐Coupling Reactions

Ni nanoparticles are highly active for conventional C─C bond‐forming reactions such as Suzuki–Miyaura, Sonogashira, and Heck coupling. For instance, Heck reactions are catalyzed by Ni nanoparticles supported on silica or carbon supports efficiently under aqueous conditions with remarkable yields and recyclability [14].

#### 1.1.6 Hydrogenation and Reduction Reactions

Magnetic Ni nanocatalysts are used in the hydrogenation of alkenes, nitro compounds, and carbonyl groups. For example, graphene oxide‐supported Ni nanoparticles have exhibited efficient catalytic hydrogenation of nitroarenes to anilines under mild hydrogen pressure [15].

#### 1.1.7 Oxidation Reactions

Ni‐based nanocatalysts have been employed in the selective oxidation of alcohols to aldehydes or ketones, typically with molecular oxygen as an oxidant, providing a green alternative to stoichiometric oxidants [13].

#### 1.1.8 C–H Activation and Functionalization

**FIGURE 3:**
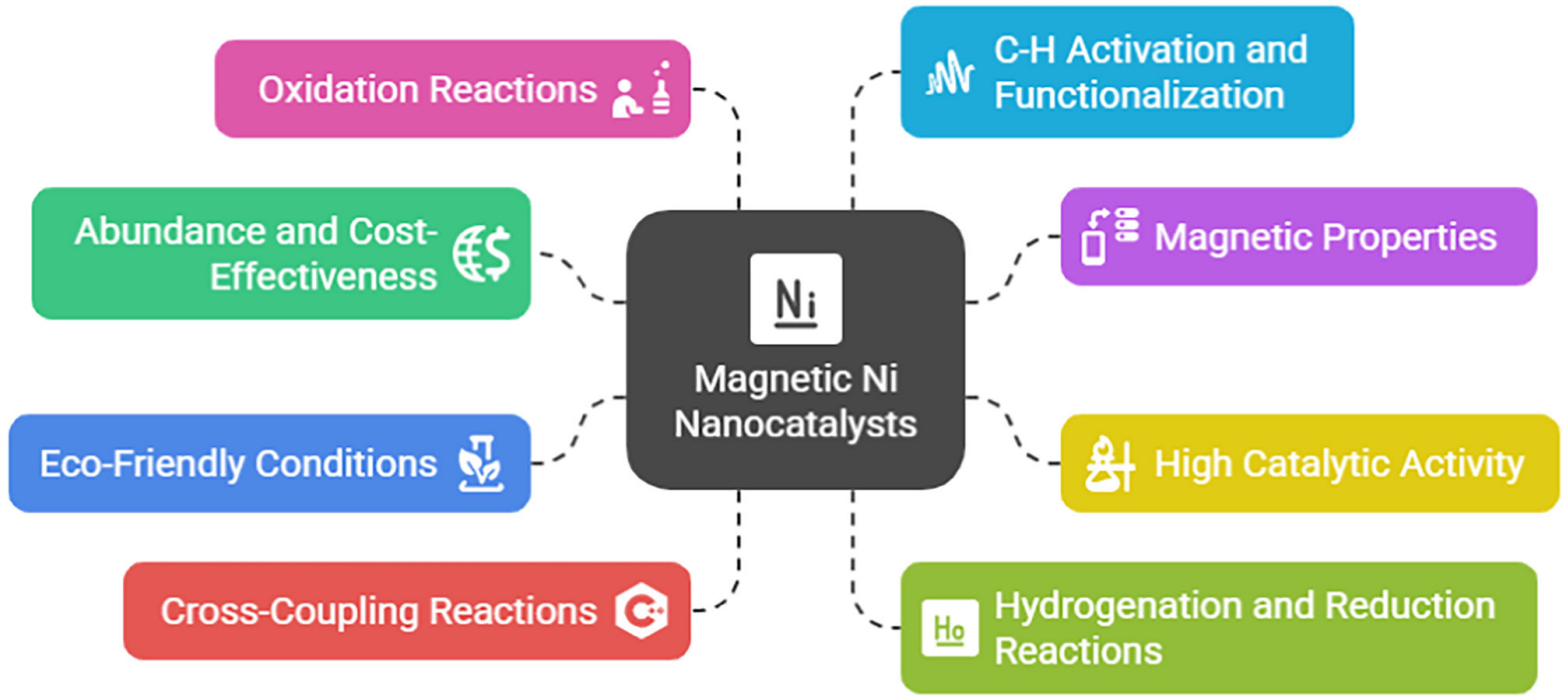
Properties and applications of magnetic Ni‐based catalysts.

Magnetic Ni catalysts have been shown recently to be able to activate inert C─H bonds on arenes, enabling direct functionalization without prefunctionalization protocols, which is extremely valuable in the simplification of synthesis pathways [16] (Figure 3).

### 1.2 Heck Reaction With Ni Nanocatalyst Applications

The Heck reaction catalyzed by Ni magnetic nanocatalysts is utilized in materials science for building π‐conjugated molecules such as trans‐stilbene [17], which is synthesized by coupling iodobenzene with ethylene [18]. Such stilbene derivatives play a vital role in the production of organic light‐emitting diodes (OLEDs), liquid crystals, and photoconductive materials due to their photophysical and electronic properties. Materials science also benefits from Heck chemistry, particularly in the assembly of π‐conjugated systems. Trans‐stilbene derivatives, prepared by Heck coupling of ethylene and iodobenzene, provide the framework in the fabrication of optical and electronic materials like OLEDs, liquid crystals, and photoconductors. These substances are highly electron‐mobile and photostable and find suitability for applications in high‐technology applications.

#### 1.2.1 Incorporation of Magnetic Nanocatalysts Into the Heck Reaction

Magnetic nickel‐based nanocatalysts are a technology‐driven mix of catalysis and nanotechnology that provides unique benefits towards chemical synthesis. Nickel is a transition metal with inherent catalytic ability, which, if complemented with small size and magnetic sensitivity, renders the resultant catalysts effective as well as recyclable. This research discusses the synthesis, characterization, and application of magnetic nickel‐based nanocatalysts in the Heck reaction to delineate their role in improving the efficiency and yield of the reaction. The main aims of this research work are to investigate the synthesis and characterization of nanocatalysts based on nickel, analyze their catalytic mechanism for the Heck reaction, and assess their performance in comparison with traditional catalysts. Additionally, the study will investigate the environmental and economic advantages of these nanocatalysts in terms of sustainability and affordability for industrial use. By highlighting these aspects, the article seeks to bring to light the possibility of magnetic nickel‐based nanocatalysts to further the science of organic synthesis. Considerable success has been realized over the past few years concerning the goals of Green Chemistry. In light of this, there has been renewed focus on more sustainable options for executing the Heck reaction, namely, the application of catalysts, nontoxic, low‐cost reagents, and renewable starting materials. The article outlines the structural/morphological features of selected case studies, along with a detailed account of the catalytic activity of magnetite‐based nanocatalysts within the field of Heck chemistry.

In 2013, Safari et al. presented the synthesis and catalytic performance of a new heterogeneous catalyst, Ni(II) loaded 1‐methyl‐3‐(3‐trimethoxysilylpropyl) imidazolium chloride ionic liquid‐supported magnetic Fe_3_O_4_ nanoparticles (IL‐Ni(II)‐MNPs) [19]. Without using expensive palladium (Pd) catalysts, the main goal was to develop an efficient and easily recoverable catalyst for the Heck reaction. There were two steps in the synthesis of this catalyst. First, coprecipitation was employed to form Fe_3_O_4_ nanoparticles, which were approximately 18–20 nm in diameter. Then, the Ni^2+^‐doped ionic liquid was used to functionalize the nanoparticles.

The superparamagnetic property of the catalyst, which allows for simpler separation via an external magnetic field, was determined via magnetometry analysis. Under the condition of the IL‐Ni(II)‐MNPs catalyst, the Heck coupling between iodobenzene and ethyl acrylate was examined. Triethylamine (Et_3_N) was identified as the most efficient base, based on optimal experiments, resulting in 97% at 100°C. With a set of aryl halides and olefins, the catalyst showed outstanding catalytic activity; aryl iodides showed exceptionally high yields. One of the notable aspects of the catalyst is its recyclability. Very stable and recoverable by magnetism, the catalyst was easily recovered and could be recycled in five successive cycles with minimal loss of yield.

**SCHEME 1:**
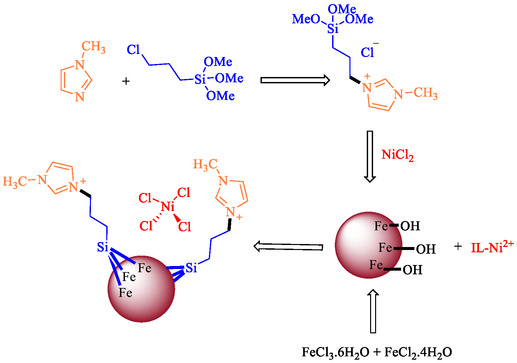
Preparation steps for fabricating IL–Ni(II)‐functionalized magnetic Fe_3_O_4_ nanoparticles.

Scheme 1 illustrates the step‐by‐step procedure for preparing magnetic Fe_3_O_4_ nanoparticles functionalized with an ionic liquid and Ni(II) ions. Magnetic Fe_3_O_4_ nanoparticles with a size of 18–20 nm are initially prepared through coprecipitation of iron(II) and iron(III) ions in a basic medium at 85°C. In the next step, this ionic liquid reacts with NiCl_2_ in acetonitrile solvent under reflux conditions for 24 h to produce the IL–Ni(II) complex. Finally, this IL–Ni(II) complex is added to the surface of Fe_3_O_4_ nanoparticles, and under mechanical stirring and in the presence of concentrated ammonia (28%) for 36 h under argon atmosphere, the immobilization and coating of the nanoparticles occur. After this process, the modified nanoparticles are separated, washed, and dried to obtain the final IL–Ni(II)–MNP nanocomposite, which exhibits desirable magnetic and catalytic properties for the Heck reaction.

They reported the catalytic performance of ionic liquid‐immobilized Ni^2+^ on magnetic Fe_3_O_4_ nanoparticles as an efficient catalyst in the Heck reaction to optimize them, systematically investigated key reaction parameters, the nature of the base, catalyst loading, and reaction temperature to establish the best conditions for the Heck coupling with their IL–Ni(II)–MNP catalyst.

**SCHEME 2:**
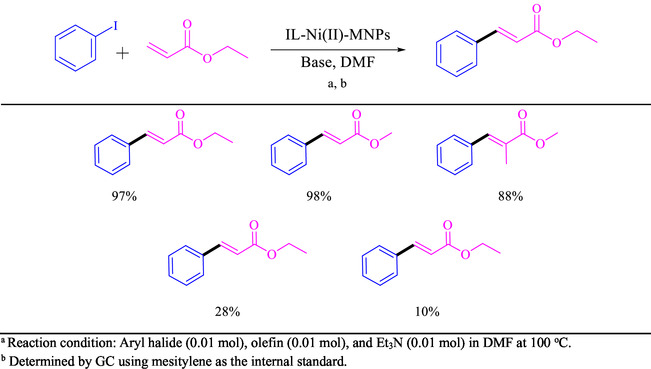
Heck coupling reaction of aryl halides with olefins catalyzed by IL–Ni(II)–MNPs.

The catalytic activity of IL–Ni(II)–MNPs was further evaluated using various aryl halides and olefins under the optimized conditions (Scheme 2). Aryl iodides bearing electron‐donating groups (e.g., methyl and methoxy) as well as unsubstituted iodobenzene exhibited excellent reactivity, affording the corresponding coupling products in 88–98% yields within 4 h. In contrast, 4‐iodonitrobenzene (electron‐withdrawing group) gave a significantly lower yield (28%), and the reaction of 4‐bromonitrobenzene proceeded sluggishly, producing only 10% of the desired product even after 10 h.

Liu et al. reported the synthesis of a palladium–NiFe_2_O_4_–graphene oxide composite magnetic material (NiFe_2_O_4_@GO–Pd) by an easy one‐pot hydrothermal route for the creation of a recoverable and efficient catalyst for the Heck reaction [20]. The synthesis route involves the dispersion of graphene oxide, prepared through a modified Hummers method, in an ethanol–ethylene glycol solution. Upon stirring, solutions of precursor NiCl_2_·6H_2_O and Fe(NO_3_)_3_·6H_2_O are added, and pH is adjusted to ~10 using aqueous ammonia, followed by the addition of PdCl_2_. The reaction mixture is loaded into a Teflon‐lined autoclave and heated at 120°C for 24 h. Such simultaneous synthesis of NiFe_2_O_4_ nanoparticles and reduction of PdCl_2_ leaves Pd and NiFe_2_O_4_ nanocrystals directly immobilized on the GO sheets without a linker. The product is rinsed and dried, giving a catalyst of about 3.5 wt% Pd. The NiFe_2_O_4_@GO–Pd catalyst was fully characterized by XRD, Raman, XPS, TEM, and VSM, all of which confirmed the existence of well‐dispersed Pd(0) and NiFe_2_O_4_ nanocrystals on graphene oxide and a superparamagnetic character (magnetization: 30.68 emu/g) that allows for facile magnetic separation. The Heck coupling of styrene and iodobenzene was used as a model to optimize the reaction conditions.

**SCHEME 3:**
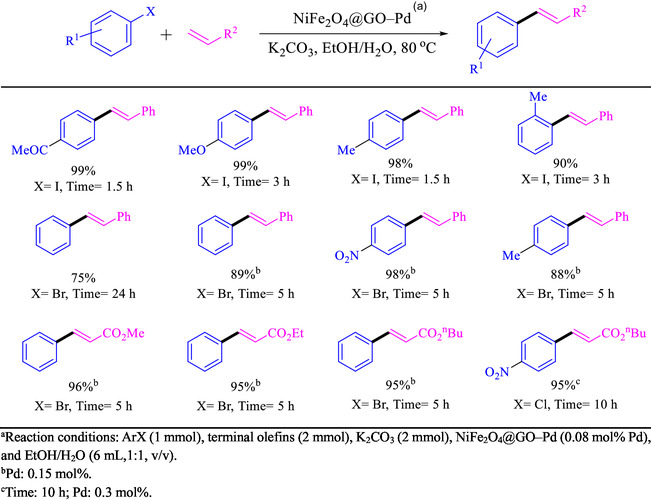
Substrate scope of the Heck reaction catalyzed by NiFe_2_O_4_@GO–Pd.

The substrate scope of the NiFe_2_O_4_@GO–Pd catalyst is shown in Scheme 3. Aryl iodides bearing both electron‐donating groups (like 4‐Me, 4‐OMe, and 2‐Me) and electron‐withdrawing ones (4‐COMe) went along quite well with styrene, giving the coupling products in about 90–99% within 1.5–3 h. By comparison, aryl bromides seemed to demand more, higher catalyst loading (0.15 mol% Pd) and longer run times (5–24 h) before moderate to high yields finally showed up. Interestingly, the catalyst also handled aryl bromides with acrylate‐type olefins (CO_2_Me, CO_2_Et, CO_2_^n^Bu) really well, producing cinnamate derivatives at 95–96% yields in around 5 h. On the other hand, aryl chlorides were not so cooperative; for instance, 4‐nitrochlorobenzene with butyl acrylate only managed 45% yield even after 10 h, despite using 0.3 mol% Pd.

Wen et al. reported a cost‐effective and environmentally friendly method for preparing water‐dispersible, magnetic, nickel‐containing, hydroxyl‐functionalized carbon nanotubes (NiCNTs‐OH) via a water‐assisted chemical vapor deposition (H_2_O‐CVD) method [21]. Upon synthesis, nickel nanoparticles are introduced at the ends of the nanotubes, and water vapor introduction introduces defects and oxygen‐containing functional groups on the walls of carbon nanotubes (CNTs), enhancing their ability to anchor metal nanoparticles. Palladium nanoparticles of size 2–4 nm are immobilized on these functionalized CNTs, and a highly dispersed, magnetically recoverable Pd/NiCNTs‐OH catalyst is obtained. The catalyst exhibits good activity in the Heck cross‐coupling reaction between aryl iodides and methyl acrylate under mild conditions, achieving high yields. Structural characterization through TEM, XRD, Raman, XPS, and magnetic measurement confirms the structure, dispersion, and recyclability of the catalyst. Density functional theory calculations also confirm the strong interaction between functionalized CNT support and Pd. The magnetically recoverable catalyst is easy to separate from reaction mixtures and can be reused several times without much loss in activity. The present work discovers an environmentally benign approach for the synthesis of stable, recoverable magnet nanocatalysts for C–C coupling reactions, with catalyst stability, recyclability, and environmental benignness benefits over traditional systems.

**SCHEME 4:**
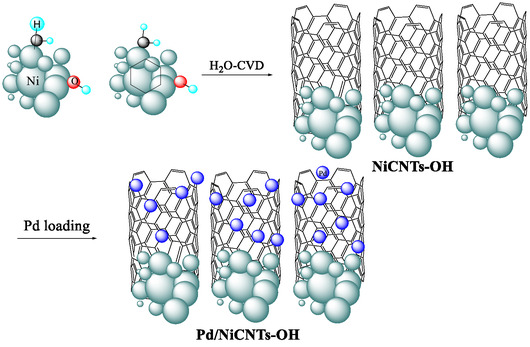
Schematic illustration of the magnetic and functional carbon nanotube in situ preparation process.

Scheme 4 shows how magnetic and functionalized carbon nanotubes, NiCNTs‐OH basically, are made using a water‐assisted CVD method. During the growth, nickel nanoparticles end up trapped inside the channels of the carbon nanotube, which gives the tubes their magnetic character, and that part happens as the cycle runs. At the same time, water vapor in the reaction also takes part in a catalytic interaction with the carbon rich surface strata of the nanotubes. Through this, oxygen functionalities like carbonyl groups and hydroxyl groups are formed. So, in the end, they obtain a strongly supported magnetic hydroxyl‐functionalized carbon nanotube carrying palladium nanowires that are well dispersed. This material acts as a solid recyclable catalyst for the Heck reaction; it offers easy recovery, higher catalytic activity, and good handling, and these are linked with specific green chemistry protocols too.

The recyclability of the Pd/NiCNTs‐OH catalyst was tested in the Heck reaction of iodobenzene with methyl acrylate under optimized conditions. After each reaction cycle, an external magnet easily separated the catalyst from the reaction mixture, thanks to its superparamagnetic properties. The recovered catalyst was washed, dried, and reused in the next runs. It maintained excellent activity over five consecutive cycles, with product yields above 95% in each cycle. A slight decrease in catalytic activity was noted only after the fifth cycle; this might be due to minimal palladium loss or gradual surface fouling. In contrast, the nonhydroxylated Pd/NiCNTs‐p catalyst had slightly lower recyclability, with yields dropping to about 90% after five cycles. These results show that the hydroxyl‐functionalized carbon nanotube support improves the stability and reusability of the palladium nanoparticles. The magnetic nickel nanoparticles embedded in the carbon nanotubes allow for easy catalyst recovery without needing filtration or centrifugation.

In addition, the impact of substituents on the catalytic activity of Pd/NiCNTs‐OH was studied using various aryl iodides with methyl acrylate. Iodobenzene provided a yield of 58% after 50 min. Among the substituted aryl iodides, those with electron‐withdrawing groups like 4‐nitro, 4‐cyano, and 4‐formyl produced yields of 51, 54, and 51%, respectively, with reaction times 90–120 min. Electron‐donating substituents such as 4‐methoxy and 4‐methyl led to yields of 48 and 57% after 50–60 min. The ortho‐substituted 2‐iodotoluene had a lower yield of 49% due to steric hindrance. The lowest yield of 29% was seen with 4‐iodoaniline after 360 min, likely because the free amino group coordinated with the palladium active sites, causing catalyst deactivation. These findings suggest that the Pd/NiCNTs‐OH catalyst works better with electronically neutral or slightly electron‐deficient aryl iodides.

**SCHEME 5:**
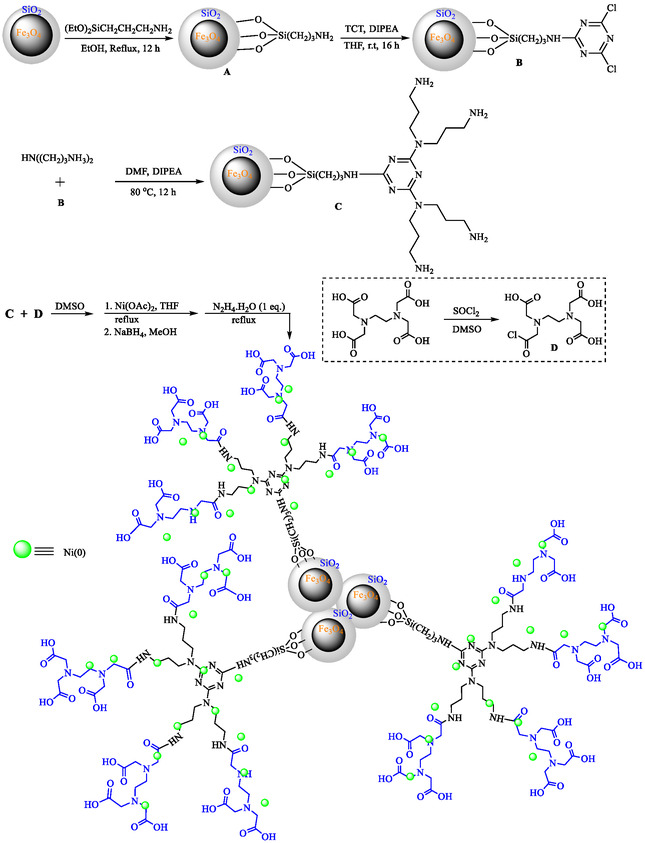
Preparation steps for fabricating nickel nanoparticles immobilized on EDTA‐modified Fe_3_O_4_@SiO_2_.

In 2020, Inaloo et al. presented the design and usage of a new air‐stable and recyclable Fe_3_O_4_@SiO_2_‐EDTA‐Ni(0) magnetically recoverable nanocatalyst for use in Suzuki–Miyaura and Heck cross‐coupling reactions [22]. This nanocatalyst is a Ni(0) nanoparticle‐immobilized platform supported on ethylenediaminetetraacetic acid (EDTA)‐functionalized silica‐coated magnetite (Fe_3_O_4_@SiO_2_) nanocatalysts. The catalyst was highly efficient in catalyzing carbon–carbon bonding formation between aryl boronic acids with aryl/heteroaryl carbamates or sulfamates, which can be called green and cheap electrophiles. Most importantly, the reaction is carried out under mild conditions without the use of any external reducing agents, with operability similar to a classical catalyst with minimal catalyst loads. In addition, isolation using an external magnet is allowed for the catalyst with at least seven times recyclability without activity loss or nickel dissolution. This research addresses the significant drawbacks of traditional palladium‐catalyzed cross‐couplings, which are costly and toxic and generate toxic waste, by offering a sustainable, affordable, and green solution that aligns with the guiding principles of green chemistry. This research highlights the significant potential of magnetic nanocatalysts in promoting green chemistry, particularly in organic synthesis applications within the pharmaceutical and agroindustry sectors (Scheme 5).

**SCHEME 6:**
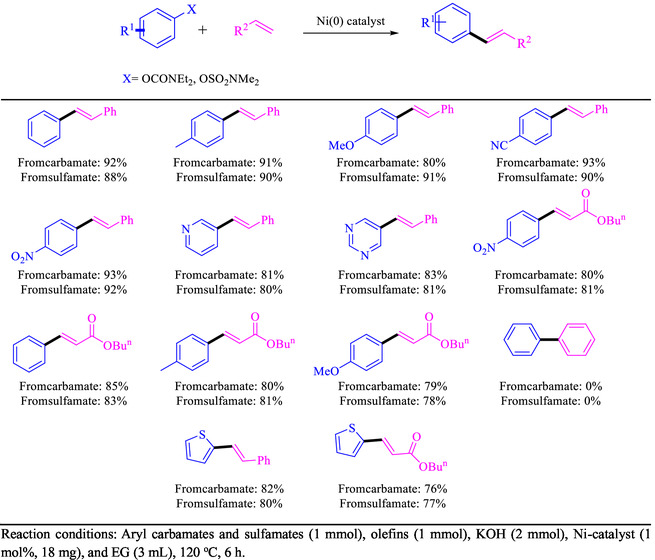
Heck cross‐coupling of aryl carbamates and sulfamates with olefins.

The catalytic behavior of Fe_3_O_4_@SiO_2_‐EDTA‐Ni(0) nanocatalyst was evaluated during Heck cross‐coupling, a range of aryl carbamates plus sulfamates, with olefins, all of it in optimized conditions (Scheme 6). Both nearly neutral and electron‐donating aryl carbamates and sulfamates ended up reacting very efficiently with styrene, giving the matching products in about 80–93% yields. For substituted styrene, those with either electron‐donating or electron‐withdrawing groups still delivered high yields, even when the aryl electrophiles were changed around a bit. When it came to aliphatic olefins like butyl acrylate, they also worked quite well with aryl carbamates that carried electron‐rich or electron‐poor type substituents, and the outcomes were fair to good, depending on the exact substrate. However, cyclohexene was different; it did not produce the expected coupling products under the same tested setup, not even after pushing the temperature up and increasing the catalyst loading. Heterocyclic carbamates and sulfamates showed a bit less reactivity compared to their aryl relatives. Taken together, these results suggest that Fe_3_O_4_@SiO_2_‐EDTA‐Ni(0) is a strongly capable catalyst for the Heck cross‐coupling of a broad set of aryl carbamate and sulfamate substrates with different olefins, using conditions that are rather mild and also more environmentally friendly than many alternatives.

**SCHEME 7:**
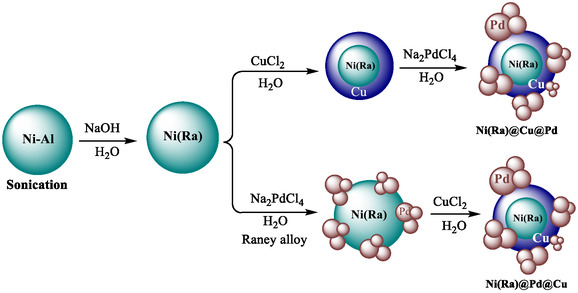
Schematic illustration of the coating procedure of Raney nickel with copper and palladium.

Bumagin in 2021 reported the synthesis of highly active, recoverable, and magnetic doped Raney nickel palladium and copper heterogeneous catalysts for the aqueous Heck coupling reaction without organic cosolvents [23]. The authors discuss some imperative issues of the field (i.e., reducing the consumption of palladium, sustainable solvents like water, and active and recoverable catalysts). Herein, the Raney nickel (89.1% Ni and 10.9% Al) was prepared and deposited with copper and palladium in two different methods to create Ni(Ra)@Cu@Pd and Ni(Ra)@Pd@Cu composite, both containing palladium. The catalysts exhibited superior activity in the Heck coupling of acrylic acid or n‐butyl acrylate with aryl halides (bromides and iodides) in water to produce high yields (97%) within 30 min at 100°C. The catalysts can be rapidly and quantitatively separated from the reaction solution by an external magnet and reused at least three times (Ni(Ra)@Pd@Cu) or four times (Ni(Ra)@Cu@Pd) without loss of activity. Atomic absorption analysis showed that a very minor percentage of the supported palladium leaches into solution under the reaction conditions, thereby rendering the catalyst stable and allowing multiple recycles. The magnetic nature of Raney nickel facilitates the easy separation of the catalyst from the reaction mixture by simply using a magnet, thus enabling its reuse in further reactions. Scheme 7 schematically illustrates the step‐by‐step synthesis of the Raney nickel‐based composite catalysts. The synthesis begins from a NiAl alloy, which is subjected to treatment with an alkaline solution and sonication to form porous Raney nickel (Ni(Ra)). The Raney nickel is further utilized as a magnetic support for the following modifications. Two different coating sequences are shown: in the first approach, copper is deposited onto the Raney nickel by treatment with an aqueous solution of copper(II) chloride, followed by deposition of palladium using sodium tetrachloropalladate, to give the Ni(Ra)@Cu@Pd composite. In the second approach, the order is inverted, palladium is deposited first, followed by copper to afford the Ni(Ra)@Pd@Cu composite.

The reaction is carried out in water, as the solvent, using 1 mol% palladium that comes from one of the polymetallic composite catalysts, for example Ni(Ra)@Cu@Pd or Ni(Ra)@Pd@Cu, and potassium carbonate (K_2_CO_3_) acts as the base. When the substrate is kind of not water compatible, meaning not soluble, the system uses K_2_CO_3_ together with tributylamine (Bu_3_N) at 20 mol% to support phase transfer. The mixture is then heated at 100°C for about 30 min, at atmospheric pressure, and this setup gives effective results. Once the reaction stops, the whole mix can be recovered rather easily with an external magnet, and it can be reused many times, without any noticeable decline in the catalytic activity.

**SCHEME 8:**
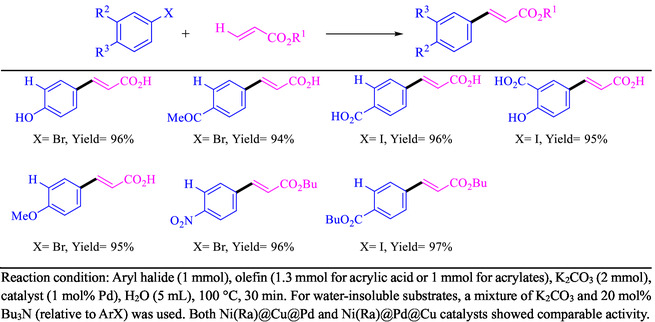
Heck coupling reaction of aryl halides with acrylic acid and acrylates catalyzed by Raney nickel‐based polymetallic catalysts (Ni(Ra)@Cu@Pd and Ni(Ra)@Pd@Cu).

The isolated products come out with high yields and a purity level around 97%, so there is no real need for further chromatographic purification. The results for the Heck coupling, run with two polymetallic catalyst variants immobilized on a Raney nickel support, specifically Ni(Ra)@Cu@Pd and Ni(Ra)@Pd@Cu, are shown in Scheme 8. The measured yields signal that the catalysts are very efficient, especially for making cinnamic acid and cinnamate derivatives, reaching about 94–97% yields under mild aqueous conditions. For those substrates that do not dissolve in water, a blend of K_2_CO_3_ and tributylamine (Bu_3_N) was applied to push the reaction forward.

General procedure for the Heck reaction catalyzed by polymetallic Ni(Ra)Cu–Pd composites a reaction mixture containing acrylic acid (1.3 mmol), aryl halide (1 mmol), K_2_CO_3_ (2 mmol) or K_2_CO_3_ with 20 mol% tributylamine (relative to ArX, for water‐insoluble substrates), and catalyst 1 or 2 (1 mol% Pd; 10 mg in 5 mL H_2_O) was vigorously stirred under reflux for 30 min (this parameter was not optimized). The progress of the reaction was monitored by TLC using hexane–diethyl ether (3:1) as the eluent. After the completion of the reaction, the mixture was diluted by the addition of water (5 mL), and the catalyst was separated by an external magnet for possible reuse. The filtrate was then warmed to 60°C and filtered hot to remove insoluble impurities. Thereafter, ethanol (10–15%, v/v) was added, and the solution was heated to about 50°C. Accordingly, 5% HCl was added dropwise with stirring until the pH changed to 2–3, leading to the precipitation of analytically pure substituted cinnamic acids as filterable precipitates. For water‐insoluble butyl cinnamates, the products were extracted with dichloromethane (3 × 5 mL). A rotary evaporator removed the solvent and excess butyl acrylate, and the raw products were purified by flash chromatography on silica gel to obtain the desired butyl cinnamates.

**SCHEME 9:**
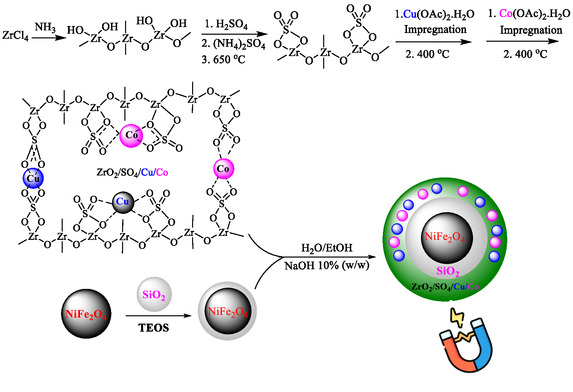
Synthesis of the NiFe_2_O_4_@SiO_2_@ZrO_2_/SO_4_^2−^/Cu/Co nanoparticles.

Alavi et al. in 2021 explored the synthesis of a new, potent, magnetically recoverable bimetallic nanocatalyst, NiFe_2_O_4_@SiO_2_@ZrO_2_/SO_4_^2−^/Cu/Co, aimed for use in palladium‐free Suzuki, Heck, and C–N cross‐coupling reactions carried out under heterogeneous conditions in water [24]. This catalyst contains a magnetic NiFe_2_O_4_ core covered with silica and sulfated zirconia, and the surface is decorated with copper and cobalt nanoparticulates, thus composing a composite that synergistically integrates the beneficial properties of both metals and allows easy magnetic separation and recyclability. The catalyst exhibited excellent activity and selectivity toward a wide array of aryl halides (particularly recalcitrant aryl chlorides), olefins, phenylboronic acids, and amines, thus allowing effective C─C and C─N bond formation under environmentally friendly and mild conditions, using water as the solvent. Crucially, it was proven that the catalyst could be used at least ten times without notable loss of efficiency or leaching of metal (Scheme 9).

**SCHEME 10:**
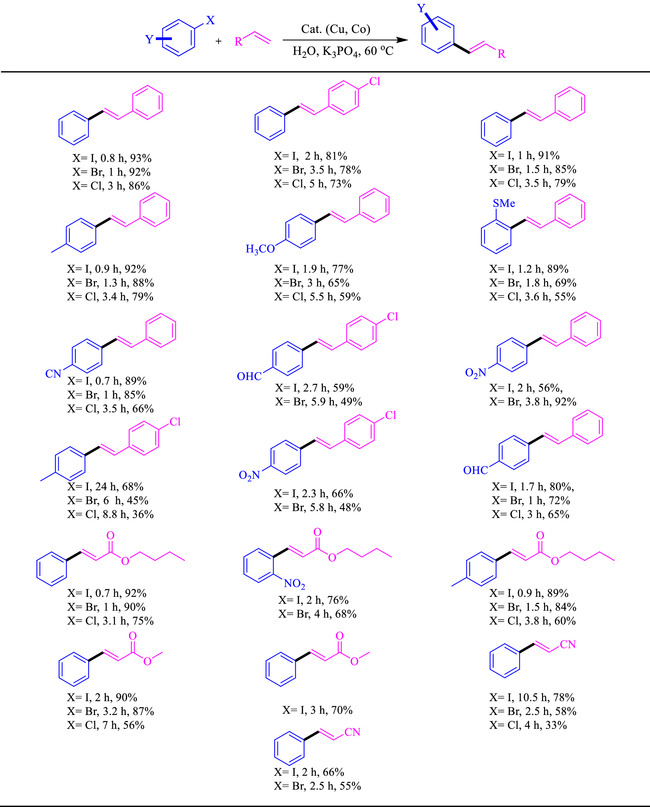
Optimization studies for NiFe_2_O_4_@SiO_2_@ZrO_2_/SO_4_^2−^/Cu/Co catalyst.

The effects of reaction parameters, including catalyst amount, temperature, base type, and solvent type, have been thoroughly studied. From Scheme 10, one can see that polar protonated solvents such as ethanol, methanol, and water perform the reaction process in equal time intervals and give much better results, hence showing the hydrophilic property of the catalyst surface. On the other hand, polar or low‐polar protonated solvents showed moderate to low performance in the reaction of aprotic or low‐polar solvents. Additional experiments were performed to check the effect of different bases, different temperatures, and different amounts of catalyst under the optimum conditions. With the help of water solvent, the highest yield was obtained at 60°C, K_3_PO_4_ being used as the base, with 3 mg of catalyst. Temperature had a role in the efficiency of the reaction as well; room temperature led to an output of just 70% in 240 min, whereas an increase in temperature to 80°C led to a yield of 96% in 50 min. An increase in temperature to reflux led to a slight decrease in yield to 90%. The optimum conditions were thus determined as catalyst (3 mg), base (K_3_PO_4_), solvent (H_2_O), temperature (80°C), and yield (96%).

Mahmoodabadi et al. in 2024. explained the synthesis and application of a new magnetically separable nanostructured catalyst, Fe_3_O_4_@WO_3_‐E‐SMTU‐Ni^II^, that allows the efficient formation of carbon–carbon bonds under eco‐friendly conditions [25]. The catalyst is synthesized by immobilization of nickel(II) ions over a magnetic core–shell structure composed of magnetite (Fe_3_O_4_) with a layer of tungsten trioxide (WO_3_) coated on it and functionalized through aminated epichlorohydrin and S‐methylisothiourea. The comprehensive characterization of the nanoparticles using a variety of techniques like FT‐IR, XRD, TEM, FE‐SEM, EDX, VSM, TGA, and ICP‐OES unequivocally confirms their spherical nature, superparamagnetic behavior, and homogeneous elemental distribution. The size of the nanoparticles ranges from 19 to 31 nm. The catalyst shows superior antibacterial activity, especially against Staphylococcus aureus. Notably, it shows exceptional catalytic performance for the Heck–Mizoroki and Suzuki–Miyaura cross‐coupling reactions and hence the effective formation of C─C bonds between a range of aryl halides (I, Br, and Cl) and alkyl acrylates or arylboronic acids. The reactions are performed efficiently under mild conditions using ethanol as an eco‐friendly solvent, a benign base derived from the aqueous solution of banana peel ash, thereby avoiding the use of toxic solvents or expensive metals in the process. The catalyst has a high loading of nickel with no metal leaching, resulting in excellent stability and recyclability for up to six cycles with only slight loss of activity. Its ease of magnetic separation, high efficiency, and eco‐friendliness make this catalyst an up‐and‐coming candidate for eco‐friendly and industrially viable organic conversions.

**SCHEME 11:**
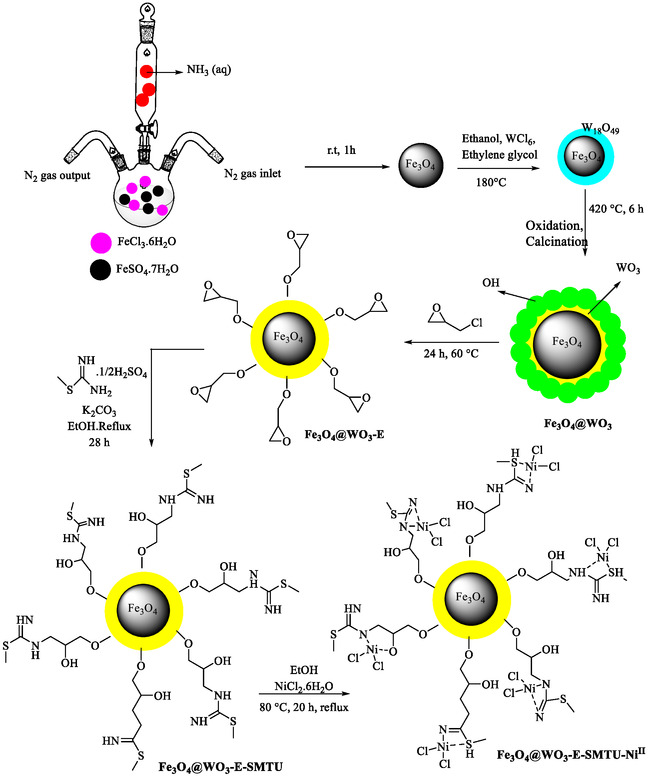
Stepwise synthesis route for the preparation of the Fe_3_O_4_@WO_3_‐E‐SMTU‐Ni^II^ nanostructure.

Scheme 11 outlines the stepwise synthesis of the Fe_3_O_4_@WO_3_‐E‐SMTU‐Ni^II^ nanostructured catalyst material. Synthesis starts with the synthesis of magnetite nanoparticles (Fe_3_O_4_) by a coprecipitation method. These Fe_3_O_4_ nanoparticles are then coated with a shell of tungsten trioxide (WO_3_), giving rise to the formation of a core–shell nanostructure (Fe_3_O_4_@WO_3_). In the next step, the composite surface is functionalized with epichlorohydrin molecules, which introduce reactive epoxide groups (Fe_3_O_4_@WO_3_‐E) onto the composite surface. In the latter step, epoxide groups were additionally functionalized using the addition of S‐methylisothiourea with a base for the deposition of aminated functionalities (Fe_3_O_4_@WO_3_‐E‐SMTU) onto the composite’s surface. Then, nickel(II) ions were immobilized on the surface‐functionalized composite material using coordination with nitrogen and sulfur donor atoms applied in the course of processing for the formation of the resultant catalyst (Fe_3_O_4_@WO_3_).

**SCHEME 12:**
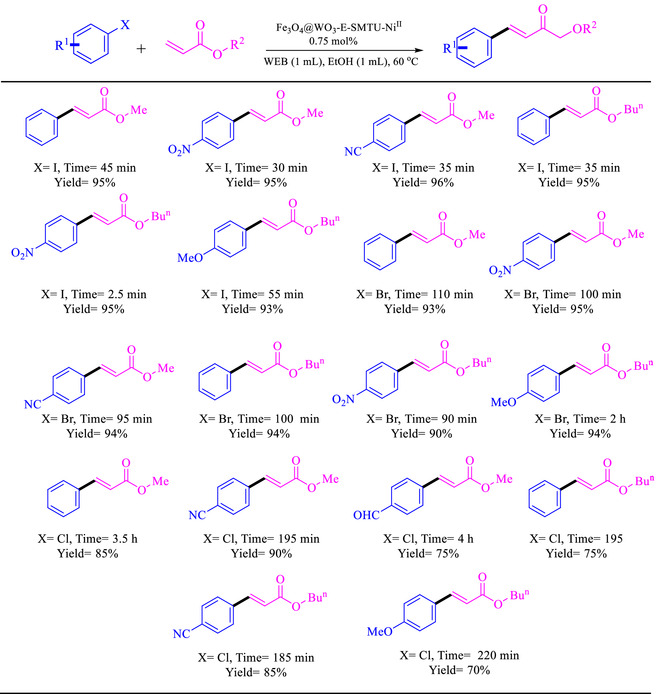
Heck–Mizoroki reaction of various aryl halides with olefins catalyzed by Fe_3_O_4_@WO_3_‐E‐SMTU‐Ni^II^.

The substrate scope of the Fe_3_O_4_@WO_3_‐E‐SMTU‐Ni^II^ catalyst was evaluated using various aryl halides and olefins under the optimized conditions (reaction conditions: aryl halide (1 mmol), olefin (1.5 mmol), K_3_PO_4_ (1 mmol), catalyst (3 mg), H_2_O (1 mL), 60°C) in Scheme 12. Aryl iodides bearing both electron‐donating (4‐OMe) and electron‐withdrawing (4‐NO_2_, 4‐CN) groups reacted efficiently with methyl acrylate and butyl acrylate, affording the corresponding coupling products in 93–96% yields within 30–55 min. Notably, 4‐nitroiodobenzene showed the highest reactivity, giving 95% yield in just 30 min with methyl acrylate and 95% yield in 2.5 min with butyl acrylate. Aryl bromides also performed well but required longer reaction times (90–110 min) to achieve comparable yields (90–95%). Electron‐withdrawing substituents on aryl bromides (4‐NO_2_, 4‐CN, 4‐CHO) facilitated the reaction, while the electron‐donating 4‐OMe group gave a slightly lower yield (93%) after 55 min. Aryl chlorides were more challenging substrates, requiring extended reaction times (3.5–4 h) and giving moderate yields (70–90%). For example, 4‐chlorobenzonitrile coupled with methyl acrylate afforded 90% yield after 195 min, while 4‐chlorobenzaldehyde gave 75% yield after 4 h. These results demonstrate that the Fe_3_O_4_@WO_3_‐E‐SMTU‐Ni^II^ catalyst is highly effective for a broad range of aryl iodides and bromides and even shows reasonable activity toward the less reactive aryl chlorides under mild and environmentally friendly conditions.

**SCHEME 13:**
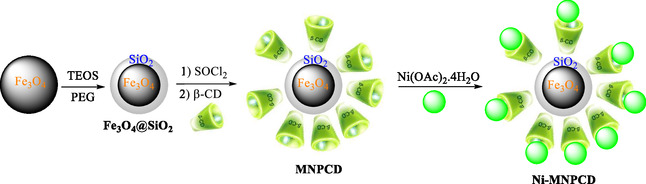
Synthetic pathway toward the preparation of Ni‐MNPCD (nickel supported on β‐cyclodextrin‐functionalized magnetic nanoparticles) catalyst.

Recently, in 2025, a study was published that described the preparation of a magnetic nickel‐containing catalyst with a polysaccharide support for the Heck reaction, reflecting the increasing focus on the use of bioderived materials as supports for sustainable catalyst design [26]. In this study, nickel‐containing species were immobilized on a carbohydrate polymer support that was functionalized with magnetic components, which facilitated easy separation and recycling of the catalyst. The catalyst showed acceptable activity under relatively mild conditions, while also enjoying the dual benefits of using renewable polymeric supports and magnetic separability. This study further underlines the flexibility of nickel‐containing magnetic catalysts and their usefulness in sustainable cross‐coupling reactions. The β‐cyclodextrin (β‐CD) magnetic nickel catalyst was synthesized using a sequential assembly strategy. First, magnetic Fe_3_O_4_ nanoparticles were prepared using a traditional coprecipitation method and then embedded into a β‐CD matrix via hydrogen bonding and coordination interactions, forming a stable magnetic biopolymer support. Due to the high density of hydroxyl groups in β‐CD, the resulting β‐CD@Fe_3_O_4_ matrix offers multiple anchoring points for metal ions. Finally, nickel ions were immobilized on the magnetic β‐CD support using coordination interactions, resulting in a recyclable nickel magnetic catalyst with high structural stability and efficient magnetic separability (Scheme 13).

**SCHEME 14:**
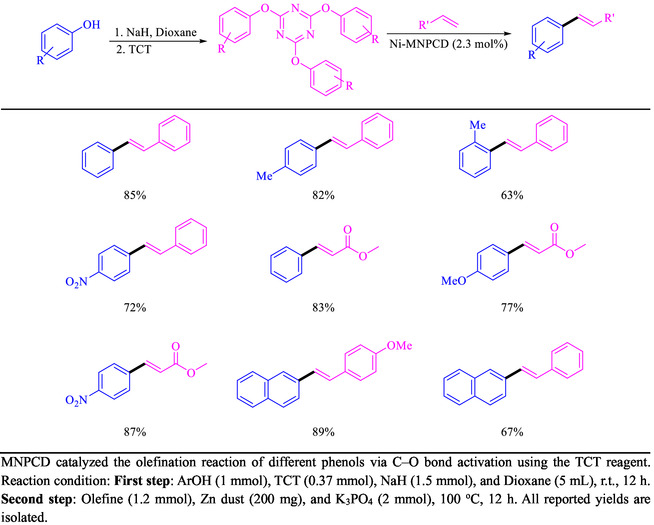
Ni‐MNPCD‐catalyzed olefination of activated phenol derivatives with styrene and methyl acrylate.

The applicability of the Ni‐MNPCD catalytic system for C─C bond formation was evaluated through the olefination of various activated phenol derivatives (Scheme 14). The reaction showed a wide substrate scope, with the desired olefinated products obtained in moderate to excellent yields (63–89%). Phenols bearing electron‐withdrawing substituents showed good reactivity, affording the corresponding products in 72–87% yields. Electron‐donating groups such as methoxy also facilitated the reaction, giving high yields of the olefinated products (77–89%). Unsubstituted phenol resulted in an excellent yield of 85%, indicating the overall efficiency of the catalytic system. The reaction conditions involved activation of the phenol with TCT (2,4,6‐trichloro‐1,3,5‐triazine) followed by coupling with styrene or methyl acrylate in the presence of the Ni‐MNPCD catalyst, Zn dust, and K_3_PO_4_ at 100°C for 12 h.

**SCHEME 15:**
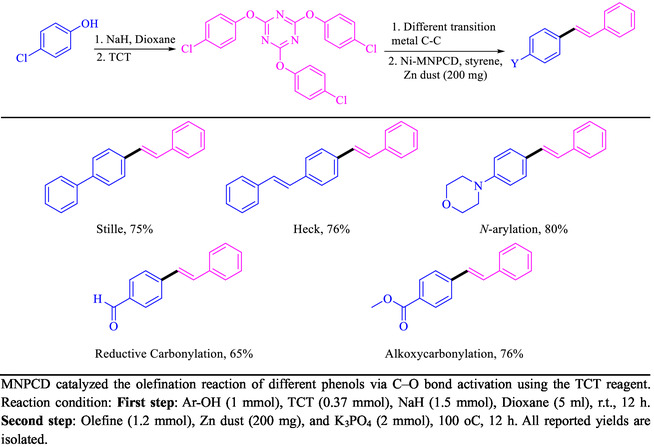
Sequential transition metal‐catalyzed functionalization and Ni‐MNPCD‐catalyzed olefination of *p*‐chlorophenol derivatives.

For an even more advanced protocol, a combination of the Ni‐MNPCD catalytic system with sequential transition metal‐mediated functionalization was explored (see Scheme 15). Here, the starting material was *p*‐chlorophenol, which, upon activation by TCT, participated in different cross‐coupling reactions catalyzed by transition metals, namely, the Stille coupling reaction (yield 75%), Heck reaction (76%), *N*‐arylation reaction (80%), reductive carbonylation (65%), and alkoxycarbonylation (76%). All of these functionalized phenols reacted with styrene in the Ni‐MNPCD‐catalyzed olefination reaction using optimized conditions. The above‐mentioned protocol demonstrates the versatility of this catalytic system because various functional groups can be attached to the phenolic structure before the final C–C bond formation step.

## 2 Comparison of the Mentioned Studies

Table 1 highlights the catalytic effectiveness of several magnetic nanocatalysts in the Heck coupling reaction at varying reaction conditions. The majority of the catalytic materials mentioned were found to have catalytic effectiveness with product yield exceeding 90%. Of these, the highest product yield was achieved using Pd/NiCNTs‐OH (99.2%) in DMF at a temperature of 100°C, while Ni‐MNPCD had the lowest catalytic effectiveness with a product yield of only 76%. In addition, the data shows that reaction effectiveness is highly dependent on the type of reaction medium and temperature, with solvents like DMF, ethanol, water, and aqueous mixture being frequently used.

**TABLE 1:** Comparison of magnetic nanocatalysts for the Heck reaction.

Entry	Magnetic nanocatalyst	Yield, %	Solvent	Temp., °C	Ref.
1	IL–Ni(II)–MNPs	97	DMF	100	[19]
2	NiFe_2_O_4_@GO–Pd	98	EtOH/H_2_O	80	[20]
3	Pd/NiCNTs‐OH	99.2	DMF	100	[21]
4	Pd/NiCNTs‐p	90.6	DMF	100	[21]
5	Fe_3_O_4_@SiO_2_‒EDTA‐Ni(0)	93	EG	120	[22]
6	Ni(Ra)@Cu@Pd	99	K_2_CO_3_/H_2_O	100	[23]
7	Ni(Ra)@Pd@Cu	97	K_2_CO_3_/Bu_3_N/H_2_O	100	[23]
8	NiFe_2_O_4_@SiO_2_@ZrO_2_/SO_4_^2‒^/Cu/Co NPs	93	H_2_O	60	[24]
9	Fe_3_O_4_@WO_3_‒E‐SMTU‐Ni^II^	95	EtOH	60	[25]
10	Ni‐MNPCD	76	DMF	120	[26]

## 3 Conclusion

This review shows that magnetic nickel‐based nanocatalysts are promising and sustainable cocatalysts alongside traditional palladium‐based catalysts in the Heck reaction. Their natural abundance, low cost, easy magnetic separation, and high catalytic activity make them attractive partners to palladium. When combined with palladium in well‐designed catalytic systems and used with green solvents like water or ethanol, these catalysts together provide high yields, outstanding selectivity, and significant reusability. They can be applied in pharmaceutical, agrochemical, and advanced material syntheses, highlighting their potential to support and enhance palladium performance in many carbon–carbon coupling processes. Although palladium remains the primary active metal, the incorporation of nickel as a cooperative component offers additional tunability and sustainability for complex couplings.

## 4 Outlook

Future directions in developing magnetic Ni‐based nanocatalysts for the Heck reaction should focus on designing better supports, like MOFs, COFs, and hierarchical porous carbons. These improvements will help with the dispersion and accessibility of active sites. We also need to work on creating stable ligands or ligand‐free systems, and expand the range of substrates to include challenging electrophiles such as aryl chlorides and sterically hindered alkenes. More in‐depth mechanistic studies using in situ spectroscopy and computation will allow for more innovative catalyst improvements. Additionally, integrating these catalysts into scalable continuous‐flow systems, using renewable energy sources in photo‐ or electro‐assisted Heck couplings, and conducting thorough life‐cycle sustainability assessments will be crucial. With ongoing interdisciplinary innovation, Ni magnetic nanocatalysts have strong potential for future industrial applications.

## Author Contributions

**Forouzan Karimi:** investigation, writing – original draft, software. **Hamideh Sarreshtehdar Aslaheh:** conceptualization, writing, review, and editing. **Ahmad Poursattar Marjani:** investigation, supervision, software, writing, review, and editing. **Kurosh Rad‐Moghadam:** supervision.

## Conflicts of Interest

The authors declare no conflicts of interest.

## Data Availability

Data sharing is not applicable to this article as no datasets were generated or analyzed during the current study.
